# The Role of Exposure Time on the Subjective Ratings of Emotional Images in Younger and Older Adults

**DOI:** 10.1007/s42761-025-00326-9

**Published:** 2025-10-31

**Authors:** Briana L. Kennedy, Jacqueline W. Tan, Tijl Grootswagers, Steven B. Most

**Affiliations:** 1 School of Psychological Science, The University of Western Australia, Perth, WA Australia; 2 The MARCS Institute for Brain, Behaviour and Development, Western Sydney University, Sydney, NSW Australia; 3 School of Computer, Data and Mathematical Sciences, Western Sydney University, Sydney, NSW Australia; 4 School of Psychology, UNSW Sydney, Sydney, NSW Australia

**Keywords:** Emotional ratings, Emotion perception, Exposure time, Positivity effect

## Abstract

Does the subjective emotionality of images change when they are only shown briefly, and are differences in the impact of emotional image duration modulated by age? The first question has broad implications for research on how emotional stimuli impact behavior, and the second question is pressing in light of evidence for age-related changes in how emotions are experienced. Here we present a study run in 2021, in which younger adults (*n* = 125) and older adults (*n* = 115) rated the emotional valence (positive vs. negative) and arousal of images that were presented for either 100 or 1,000 ms. Emotion ratings were strongly correlated between image durations, although both age groups rated emotional images as less extreme in valence and arousal when shown for less time. Older adults were affected by exposure time more than younger adults and generally rated images more positively. An exploratory analysis examined whether various visual features explained differences between fast and slow ratings. Our findings shed light on how emotional stimuli are experienced at fast speeds and how age exacerbates exposure time effects, while highlighting considerations when showing emotional stimuli at varying rates.

**Supplementary Information:**

The online version contains supplementary material available at 10.1007/s42761-025-00326-9.

Emotional stimuli can have striking effects on behavior, even when only briefly glimpsed. For example, in an “emotion-induced blindness” task, a quick flash of an emotional image impairs the detection of targets that appear immediately afterwards (e.g., Kennedy & Most, 2015a, 2015b; Most et al., 2005; Wang et al., 2012). Tasks with such fleeting exposures provide insights into fast-acting mechanisms of emotional processing, although less clear is how people *subjectively experience* emotional images when they are shown at fast speeds. In the current study, we investigated how image exposure time influences emotional ratings.

It remains an open question how quickly emotional meaning can be extracted from emotional images (e.g., Costa et al., 2014; Diano et al., 2017; Grootswagers et al., 2017; Maljkovic & Martini, 2005; Pessoa & Adolphs, 2010; Stein et al., 2014). Evidence suggests that it can happen quickly; emotional stimuli can affect behavior at fast speeds (e.g., Wang et al., 2012) and brain decoding methods can detect emotional properties of an image within hundreds of milliseconds (e.g., Grootswagers et al., 2017). However, effects of emotion can be observed without participants’ awareness of emotional stimuli (e.g., Diano et al., 2017; Pessoa & Adolphs, 2010), suggesting that effects on behavior may differ from experiences. Several theories of emotional experiences are also grounded in their temporally unfolding nature; for example, the process model of emotion regulation (Gross, 1998, 2002, 2015) assumes stages that take time to unfold, and constructivist theories (e.g., Barrett, 2017) imply that time may be required for such construction to take place.

Beyond theory, knowing how exposure times impact image ratings has practical implications. Researchers who show emotional images at fast rates (including us) tend to use emotional stimuli from established image databases (Lang et al., 2008; Marchewka et al., 2014), choose images based on normative image ratings of valence (i.e., how positive or negative) and arousal (i.e., how activating or dull), and often present images at rates much faster than they had been presented during the ratings themselves (e.g., 6 s, Bradley & Lang, 1994; Lang et al., 2008; or until a rating response is made, Marchewka et al., 2014). This approach assumes the images would be rated with the same emotional intensity when shown only briefly, but this assumption constitutes a large disconnect in the literature.

Fast exposure times of emotional stimuli may especially affect older adults. Older adults demonstrate slower processing speed than younger adults in a variety of tasks (Ebaid & Crewther, 2019; Jenkins et al., 2000; Salthouse, 2010). Older adults also tend to show a positivity bias to favor positive over negative emotions compared to younger adults and relevant theories often implicate mechanisms relevant to time, such as age-related emotion regulation strategies that may take time to enact (Barber & Kim, 2022; Charles et al., 2003; Mather & Carstensen, 2005). If time is involved in the way older adults prioritize emotions, a much shorter exposure time may especially change the way they rate emotional images.

An indication that exposure duration affects emotion ratings comes from contradictory findings on age differences: compared to younger adults, some rating studies show that older adults rate images more positively (Neiss et al., 2009; Neta & Tong, 2016) whereas some others show that older adults rate images less positively (Backs et al., 2005; Grühn & Scheibe, 2008). Although the reasons for such disparity are unclear, a possibility is that they are due to key design differences in the rating tasks (see also Reed et al., 2014). In the studies listed above, those that found that older adults rated images more positively than younger adults had participants view images for a short, set time and had participants make their ratings after seeing them: Neiss and colleagues showed images for two seconds, and Neta and Tong showed images for 500 ms (unmasked; Neiss et al., 2009; Neta & Tong, 2016). In contrast, the studies that found that older adults rated images less positively than younger adults had participants view images for a lengthy amount of time: Backs and colleagues showed images for 20 s; Grühn and Scheibe had participants progress at their own pace (Backs et al., 2005; Grühn & Scheibe, 2008). Thus, studies that showed an age-related bias to rate images more positively presented images at a set pace and for a shorter amount of time than similar studies that found older adults rated images less positively, indicating that the exposure time may play a role especially in the way older adults rate emotional images.

A common concern when images are shown quickly is that participants may not be affected by emotion necessarily, but instead by properties of the images used in that research, such as color, luminance, or spatial frequency. To control for this, researchers often try to choose images that clearly depict emotional content and make comparisons with emotionally neutral images with otherwise similar content and complexity (e.g., Kennedy & Most, 2015a; Most et al., 2005). Some research indicates that image properties do not play a substantial role in the experience of emotions (Maljkovic & Martini, 2005), while others indicate that some properties, such as high spatial frequencies, are related to the detection of certain emotional stimuli (Stein et al., 2014). To investigate this, in addition to examining image ratings at different durations, we also examined whether different image properties could contribute to differences in ratings.

The aim of this study was to determine if exposure time and age changed the way individuals subjectively rated images for their emotionality. Younger and older adult participants rated emotionally positive, negative, and neutral images along the dimensions of valence and arousal. For each participant, half of the images were presented for a shorter duration (100 ms) and the other half were presented for a longer duration (1,000 ms). We predicted that ratings would be mostly similar at fast and slow rates, but that ratings of emotional images would be blunted (i.e., rated as less emotional) when shown quickly compared to when shown for a longer amount of time, especially in older adults. We approached our questions related to image properties in a more exploratory way to examine whether image features could predict the difference in ratings at fast versus slow exposures.

## Method

### Participants

Two hundred sixty-two participants originally took part in our study. Participants were deemed eligible for the study if they were between 18–30 or 60–80 years of age, had normal or corrected-to-normal vision, had the required technical hardware, and were not taking beta-blocker medication. Data from thirteen participants were excluded from data processing for failure to meet age requirements and an additional nine participants (eight younger adults and one older adult) were excluded for reporting not having normal or corrected-to-normal vision. Thus, analyses were conducted with data from 125 younger adults (18–30 years of age, *M* = 19.7 years, *SD* = 2.4 years; 80 female, 42 male, 3 preferred not to state) and 115 older adults (60–80 years of age, *M* = 65.7, *SD* = 4.7; 57 female, 58 male; 61 retired, 54 employed).

Younger adults were undergraduate students from the University of Western Australia and were recruited through the university’s SONA research participation system. Older adults were recruited through CloudResearch TurkPrime (Litman et al., 2017), connected to the Amazon Mechanical Turk Platform. All older adults in the experiment were recruited from the USA.

Our sample size was chosen to be able to observe small effects in our data, since the role of exposure time on emotionality ratings has never previously been examined. We aimed to recruit at least 120 participants per age group, to allow for possible exclusions to leave at least 100 per group in the final sample; our final sample was larger than the goal for each group because we batched our recruitment on a week-by-week basis. Our final sample size of 240 participants enabled observation of small effects in our predicted effects, including an age × exposure time within-between interaction (*f* =.03) and exposure time × valence within factors interaction (*f* =.18), with α =.05 and power (1 – *β*) = .90 (calculated with G*Power; Faul et al., 2007).

Younger adults received course credit for completion of the experiment, and older adults were compensated $5USD. The experiment took approximately 30 min to complete. Ethics approval was granted by the UWA Human Ethics Committee (protocol 2021/ET000232, “Rating images for their emotionality”).

### Materials

#### Stimuli

Stimuli were colored images presented as 320 × 240-pixel stimuli. This included 32 positive, 32 neutral, and 32 negative images selected from the IAPS and NAPS databases (Lang et al., 2008; Marchewka et al., 2014). A list identifying the specific images that we used in the study is available in our supplemental material: https://osf.io/vxuf8. Based on previous emotion-attention studies, we selected negative and positive images that were high in arousal, and the selected neutral images were low in arousal, based on the valence and arousal rating norms provided (valence: *negative* = 1 to *positive* = 9; arousal; *low arousal* = 1 to *high arousal* = 9). Positive images depicted scenes such as erotica or extreme sports, negative images depicted scenes such as violent acts or medical injuries, and neutral images depicted scenes with people with neutral facial expressions or participating in everyday activities like playing chess. See Table 1 for valence and arousal means and 95% confidence intervals for each of the image categories.

**Table 1:** Means and 95% Confidence Intervals of valence and arousal ratings from image databases

Image type	Valence		Arousal	
	*M*	*95% CI*	*M*	*95% CI*
Positive	7.36	[7.17, 7.56]	5.66	[5.32, 6.00]
Neutral	5.30	[5.08, 5.53]	3.31	[3.14, 3.49]
Negative	2.52	[2.27, 2.77]	6.40	[6.22, 6.57]

Database-provided valence ratings for the images (Lang et al., 2008; Marchewka et al., 2014) confirmed that negative images were more negative in valence than both the neutral, *t*(31) = 19.59, *p* <.001, *d*_*z*_ = 3.46, and positive images, *t*(31) = 40.62, *p* <.001, *d*_*z*_ = 7.18, and positive images were more positive than neutral images, *t*(31) = 14.13, *p* <.001, *d*_*z*_ = 2.50. Negative and positive images were more arousing than the neutral images, *t*(31) = 23.59, *p* <.001, *d*_*z*_ = 4.17, *t*(31) = 15.73, *p* <.001, *d*_*z*_ = 2.78, and negative images were also more arousing than the positive images, *t*(31) = 3.48,* p* =.009, *d*_*z*_ = 0.62.

In addition to emotional images, fifty mask images were used in the experiment. These were created by using a Jigsaw Puzzle program in Matlab (Ouseph, 2020). Fifty landscape scenes were separated into 30 vertical and 30 horizontal sections and those sections were scrambled to create mask images similar to scrambled stimuli used by Most et al. (2005).

#### Image Rating Task

**Fig. 1:**
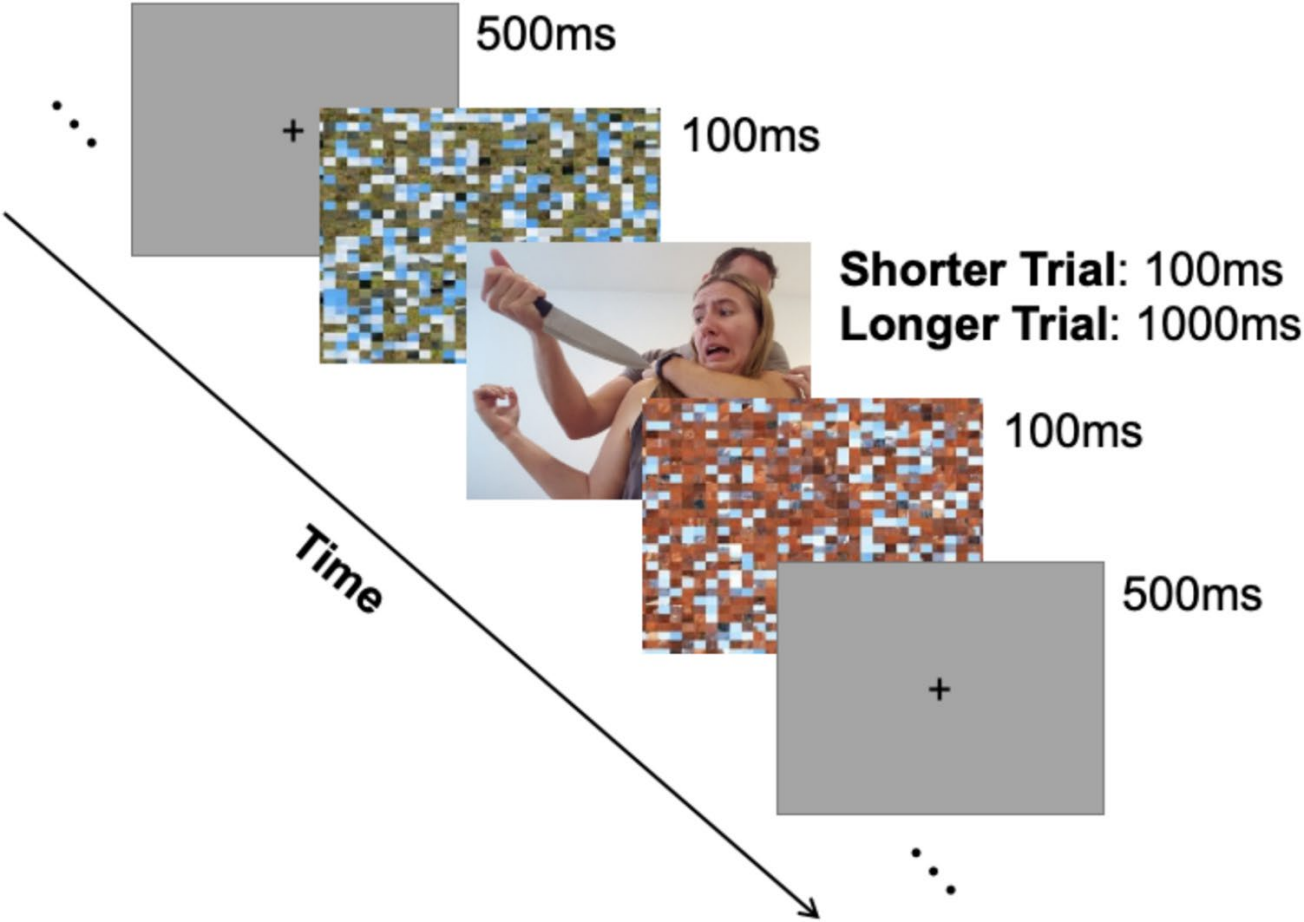
Example trial for the image rating task. *Note.* Images and masks were colored images. The critical difference between trial types was the image type and exposure time of each image. Note that the image depicted here is for demonstration purposes and was not part of the experimental stimulus set.

The image rating task consisted of 96 trials. In each trial, participants saw a fixation cross for 500 ms, a mask image for 100 ms, and were then presented with a positive, neutral, or negative image that was shown for either 100 ms or 1,000 ms (see Fig. 1). We carefully chose the times of 100 ms and 1,000 ms. Both durations are commonly used in emotion-attention research, making them well-suited for comparison and generalization across research designs in the greater emotion-attention literature (e.g., Balsamo et al., 2020; Bocanegra & Zeelenberg, 2009; Chapman et al., 2019; Most et al., 2005). For example, Bocanegra and Zeelenberg (2009) found enhanced perception 1,000 ms after an emotional stimulus, compared to perceptual detriments commonly found 100 ms after an emotional stimulus (e.g., Most et al., 2005), indicating that these shorter and longer exposure times can engage different affective mechanisms in perception. Additionally, while the tenfold difference in exposure time provided a strong variation in temporal dynamics, both were still appropriate for the task structure, which was important to examine effects of exposure time rather than differing task demands. This allowed the longer duration to be short enough to avoid disengagement from the task or extended strategic regulation to occur while long enough to be well beyond the time required for the visual processing of an image (e.g., Thorpe et al., 1996). Comparing these two durations thus enabled us to probe whether the subjective experience of emotions differed when time was constrained to a brief versus extended time exposure, while maintaining similar task demands between the conditions.

After the image was presented, another mask was shown for 100 ms, followed by another fixation cross for 500 ms. Participants then rated the emotionality of the presented image using their mouse on dropdown menus for valence (“*Valence: Did you find the image to be negative or positive?*”; 1 = *negative* to 9 = *positive*) and arousal (“*Arousal: Did you find the image to be low activating/dull or highly activating/intense?*”; 1 = *low arousal* to 9 = *high arousal*). They were also asked to indicate on a four-point scale how well they saw the image (“*How well did you SEE the image?*”; 1 = *definitely did not see* to 4 = *definitely saw*). This allowed us to identify trials when participants definitely did not see an image to exclude ratings on these trials. There was no time limit for participants to respond. After responses were submitted, the next trial began automatically. Trial types were presented in a quasi-randomized manner, such that the exposure time and emotional image type could not be predicted on any given trial and all participants saw them in a computer randomized, unique order.

To ensure that any differences from exposure time was not from the specific content of the images, we counterbalanced images between participants. There were two sets of images, such that that half of participants saw one set of images presented for 100 ms and the other set for 1,000 ms, and the other half of participants saw the same images but with images assigned the opposite exposure times.

To get used to the task, participants completed six practice trials; two with negative images, two with neutral images, and two with positive images. All participants saw the same six practice images, and these images were not used in the experiment but were similar to other images in those categories. Half of the practice trials were 100 ms and the other half were 1,000 ms. Images and trial speed were presented in an otherwise pseudo-random order, such that participants could not predict the next trial type. Practice trials were an opportunity for participants to get used to the task and data from the practice trials were not analyzed.

### Procedure

All participants completed the experiment online and on their own computer. The experiment was administered using Inquisit 6 software (Inquisit6, 2021). After providing consent, participants completed a short demographic questionnaire, and then completed the image rating task. At the end of the study, participants were debriefed about the goals of the study. Data were collected between April and July 2021.

### Image Properties

To examine image properties, we calculated statistics for each of the 96 images in the experiment across dimensions of color, spatial frequency, entropy, luminance, and root mean square (RMS) contrast. We used the Natural Image Statistical Toolbox (Bainbridge & Oliva, 2015) to estimate the average red, green, and blue color of each image, along with the spatial frequency energy (Ef) for each image in 10% (Ef10), 30% (Ef30), 50% (Ef50), 70% (Ef70), and 90% (Ef90) frequency bands and quality high frequency (QhF; sampled in the 90% band). Spatial frequency refers to the distribution of energy across space in an image, such that low spatial frequencies refer to gradual change across an image (e.g., larger areas) whereas high spatial frequency refers to rapid changes across an image (such as edges). In addition, we included image entropy (a statistical measure of randomness that can be used to characterize the texture of the input image; calculated using Matlab’s *entropy* function), mean luminance (i.e., the mean across all image pixels), and RMS contrast (i.e., the standard deviation across all image pixels).

Along with individual image features, we also submitted images to two pretrained convolutional neural networks (CNNs): Memnet and AlexNet. Memnet estimates the “memorability” of each image based on prior training on other large image databases — in other words — it estimates how well individuals would be able to remember a particular image based on prior training (Khosla et al., 2015). We also aimed to capture a more complete estimation of image properties, using AlexNet, a convolutional deep neural network with eight layers inspired by the hierarchical structure of visual processing in the brain (Krizhevsky et al., 2017). For each image, we extracted activation profiles for every convolutional (conv) and fully connected (fc) layer in AlexNet, specifically convolutional layers 1 (conv1), 2 (conv2), 3 (conv3) 4 (conv4), and 5 (conv5), and fully connected layers 6 (fc6), 7, (fc7), and 8 (fc8). As the activation profiles are high-dimensional, we transformed these using PCA and used the activation score on the first component, resulting in one value per image per layer.

In sum, for our analyses related to image properties, each image had 21 measurements: red color average, green color average, blue color average, Ef10, Ef30, Ef50, Ef70, Ef90, QhF, entropy, mean luminance, RMS contrast, memorability, conv1, conv2, conv3, conv4, conv5, fc6, fc7, and fc8.

### Transparency and Openness

We report how we determined our sample size, all data exclusions, all manipulations, and all measures in the study. The experimental program, anonymized raw data, and summarized data are available on OSF: https://osf.io/vxuf8. Images from the IAPS (Lang et al., 2008) and NAPS (Marchewka et al., 2014) image databases were used in the experiment and are thus not provided, but image names are listed in the experimental program and in the datafiles available. Data were analyzed using SPSS 26, Matlab 2023b, and R (R Core Team, 2021) and figures were made using the package *ggplot2* (Wickham, 2016). All analyses were run as planned except as noted. Participants were made aware in the consent form that anonymized data could be made publicly available via the Internet or open database for scientists to use in future work. This study was not pre-registered.

## Results

### Data Screening

Per our planned analyses, we removed trials when participants reported they “definitely did not see” the image in analyses unless specifically noted below. Since image ratings were our primary dependent variable, this was to avoid possible invalidation of the ratings. After removing these trials, an average of 88.36 of the 96 total trials were retained for each of the younger adults (*SD* = 6.47 trials; *range* = 60–96 trials), and an average of 86.83 of the 96 total trials were retained for each of the older adults (*SD* = 7.51 trials; *range* = 63–96 trials). However, for completeness, we include those trials in our major analyses in the *Supplementary Material,* which essentially replicate all results listed below.

ANOVA results were interpreted with Greenhouse–Geisser adjusted values when sphericity assumptions were violated (Gignac, 2019). Bonferroni corrections were applied to *t*-tests to account for multiple comparisons.

### Overall Correlation Between Short- and Long-Duration Ratings

**Fig. 2:**
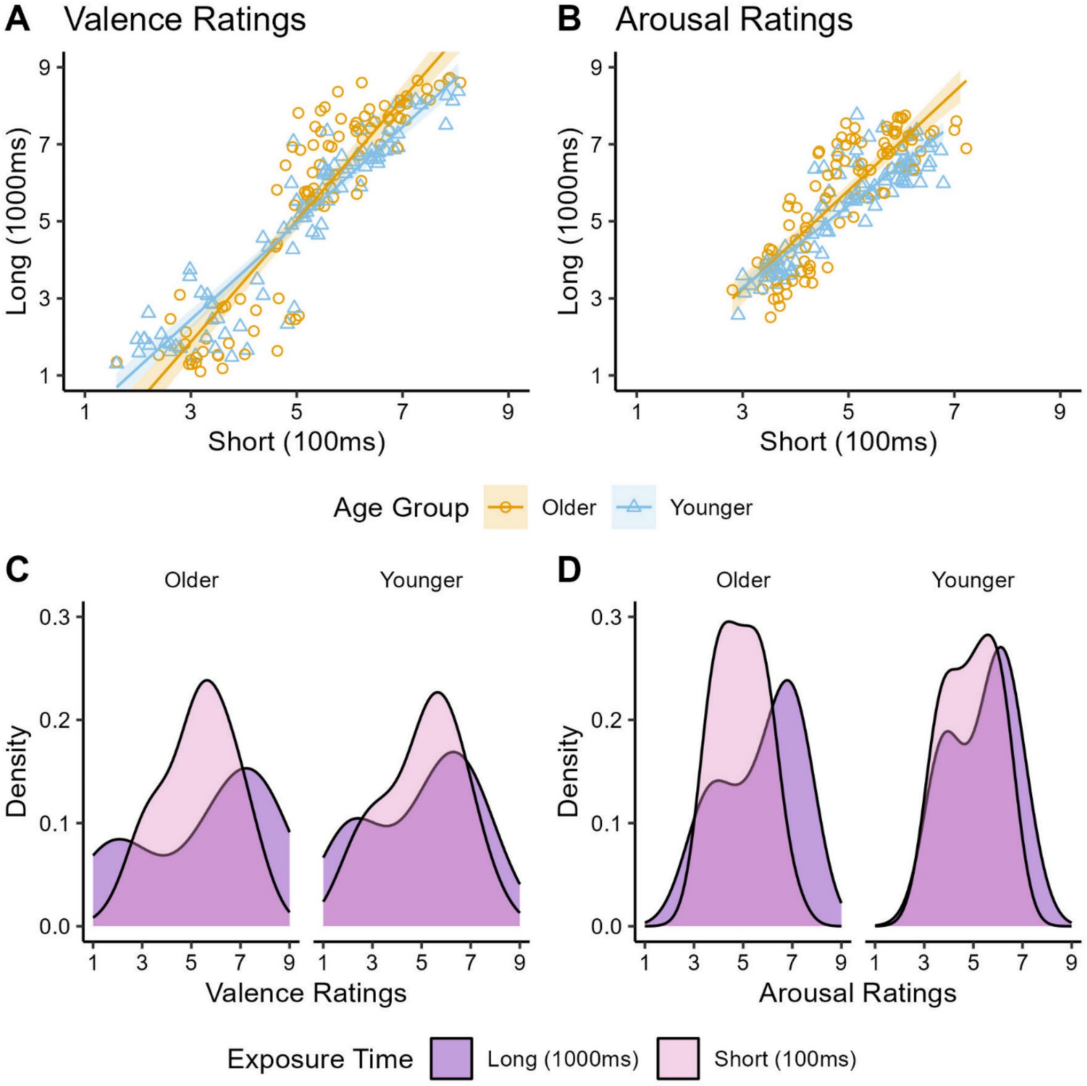
Valence and arousal ratings for all images at long and short exposures. *Note.* Younger and older adults rated images shown for long (1,000 ms) and short (100 ms) exposure times on the dimensions of valence and arousal on scales from 1 to 9. On long (1,000 ms) versus short (100 ms) exposure times in both young adults and older adults, there was a strong correlation in (A) valence ratings and (B) arousal ratings. Density plots illustrate that the spread (i.e., intensity) of ratings was greater in both younger and older adults on long (1,000 ms) versus short (100 ms) image durations in both (C) valence ratings and (D) arousal ratings.

We first wanted to determine how strongly ratings compared between shorter and longer viewing durations overall. To do this, we averaged the valence and arousal ratings for each image — regardless of image type — when it was viewed at 100 ms and 1,000 ms in younger and older adults groups separately. For both durations of 100 ms and 1,000 ms, Pearson correlations revealed that valence and arousal ratings were highly correlated in both younger adults (valence: *r* =.934, *p* <.001; arousal: *r* =.887, *p* <.001) and older adults (valence: *r* =.901, *p* <.001; arousal: *r* =.836, *p* <.001; see Fig. 2). Thus, there was a strong relationship between ratings for images shown at short and long durations in both age groups. Density plots of the ratings further indicated that in both older and younger adults, the spread of ratings was tighter in the short exposure time (100 ms) compared to long exposure time (1,000 ms), and that the long exposure time took a more bimodal shape. This indicates that participants tended to use a greater spread of ratings when they saw images for a longer amount of time, and that especially arousing negative and positive images were rated as more neutral when shown for a shorter amount of time.

### Difference in Ratings by Stimulus Duration and Image Types

We next wanted to examine how valence and arousal ratings of specific emotion image type categories (negative, neutral, and positive) compared, overall, between short (100 ms) and long (1,000 ms) exposures. Mean valence and arousal ratings for each image type are presented in Table 2.

**Table 2:** Means and 95% Confidence Intervals of valence and arousal ratings

Image type	Exposure time	Valence ratings				Arousal ratings			
		Younger adults		Older adults		Younger adults		Older adults	
		*M*	*95% CI*	*M*	*95% CI*	*M*	*95% CI*	*M*	*95% CI*
Positive	100 ms	6.63	[6.48, 6.77]	6.73	[6.58, 6.88]	5.34	[5.11, 5.57]	5.38	[5.15, 5.62]
	1,000 ms	7.12	[7.00, 7.25]	7.94	[7.81, 8.07]	5.76	[5.52, 5.99]	6.34	[6.09, 6.58]
Neutral	100 ms	5.37	[5.24, 5.50]	5.69	[5.56, 5.83]	3.67	[3.44, 3.90]	3.81	[3.56, 4.05]
	1,000 ms	5.53	[5.40, 5.67]	6.38	[6.24, 6.52]	3.75	[3.51, 4.00]	3.77	[3.52, 4.03]
Negative	100 ms	3.06	[2.90, 3.23]	3.55	[3.38, 3.73]	5.85	[5.59, 6.10]	5.55	[5.28, 5.81]
	1,000 ms	2.28	[2.14, 2.42]	2.09	[1.95, 2.24]	6.52	[6.25, 6.79]	6.96	[6.68, 7.25]

**Valence.** A 3 (image type: positive, neutral, and negative) × 2 (exposure time: 100 ms, 1,000 ms) × 2 (age: younger and older) ANOVA of valence ratings revealed a significant main effect of image type; in general, negative images were rated as most negative and positive images were rated most positive, *F*(1.51, 358.24) = 2849.06, *p* <.001, *η*^*2*^_*p*_ =.92. There was also a main effect of exposure time, *F*(1, 238) = 4.67, *p* =.032, *η*^*2*^_*p*_ =.02; in general, images shown for 1,000 ms were rated as more positive than images shown for 100 ms, although planned *t*-tests revealed that negative images were rated as more negative and positive images were rated as more positive (see below). The main effect of age was significant; older adults rated images as more positive than younger adults, *F*(1, 238) = 36.29, *p* <.001, *η*^*2*^_*p*_ =.13. This, along with a significant image type × age interaction, revealed that older adults rated images as more positive in valence than younger adults, *F*(1.51, 358.24) = 7.13, *p* =.003, *η*^*2*^_*p*_ =.03, consistent with an age-related positivity bias pattern in the ratings generally.

A significant image type × exposure time interaction, *F*(1.85, 440.22) = 703.76, *p* <.001, *η*^*2*^_*p*_ =.75, revealed that the impact of exposure duration was different for different image types (see *t*-tests below). A significant exposure time × age interaction, *F*(1, 238) = 14.96, *p* <.001, *η*^*2*^_*p*_ =.06, and image type × exposure time × age interaction, *F*(1.85, 440.22) = 91.97, *p* <.001, *η*^*2*^_*p*_ =.28, further indicated that the impact of exposure time differed between younger and older adults; compared to younger adults, older adults were more impacted by differences in exposure time when rating valence.

Planned *t*-tests revealed that in both younger and older adults, positive images were rated as less positive when observed for 100 ms compared to 1,000 ms (younger adults: *t*(124) = 12.50, *p* <.001, *d*_*z*_ = 1.12; older adults:* t*(114) = 20.09, *p* <.001. *d*_*z*_ = 1.87). Similarly, negative images were rated as less negative when observed for 100 ms compared to 1,000 ms (younger adults: *t*(124) = 15.20, *p* <.001, *d*_*z*_ = 1.36; older adults: *t*(114) = 19.63, *p* <.001, *d*_*z*_ = 1.83). Unexpectedly, both younger and older adults rated neutral images as less positive when shown for 100 ms compared to 1,000 ms (younger adults: *t*(124) = 3.42, *p* =.005, *d*_*z*_ = 0.31; older adults: *t*(114) = 10.91, *p* <.001, *d* = 1.02). Together, this indicated that overall, younger and older adults rated emotional images as less extreme in valence, and neutral images as more negative, when seen briefly.

**Arousal.** A 3 (image type: positive, neutral, and negative) × 2 (exposure time: 100 ms, 1,000 ms) × 2 (age: younger and older) mixed ANOVA of arousal ratings revealed a main effect of image type; negative and positive images were rated as more arousing than neutral images, *F*(1.56, 370.67) = 503.39, *p* <.001, *η*^*2*^_*p*_ =.68. The main effect of exposure time was also significant, with images shown for 1,000 ms rated as more arousing than images shown for 100 ms, *F*(1, 238) = 328.60, *p* <.001, *η*^*2*^_*p*_ =.58. The main effect of age was not significant, *F*(1, 238) = 1.14, *p* =.287, *η*^*2*^_*p*_ <.01, nor was the image type × age interaction, *F*(1.56, 370.67) = 1.39, *p* =.248, *η*^*2*^_*p*_ < 0.01, indicating that younger adults and older adults did not rate the arousal of images differently overall.

The image type × exposure time interaction was significant; overall participants rated the arousal of different image types differently when presented for 100 ms compared to 1,000 ms, *F*(1.91, 455.38) = 141.39, *p* <.001, *η*^*2*^_*p*_ =.37. The exposure time × age interaction was also significant, *F*(1, 238) = 35.93, *p* <.001, *η*^*2*^_*p*_ =.13, as was the age × exposure time × image type interaction, *F*(1.91, 455.38) = 26.56, *p* <.001, *η*^*2*^_*p*_ =.10. Like valence ratings, older adult arousal ratings were more impacted by differences in exposure time than younger adults.

In younger and older adults, both positive images (younger: *t*(124) = 7.53, *p* <.001, *d* = 0.67; older: *t*(114) = 12.63, *p* <.001, *d*_*z*_ = 1.18) and negative images (younger: *t*(124) = 9.49, *p* <.001, *d* = 0.85; older: *t*(114) = 18.19, *p* <.001, *d*_*z*_ = 1.70) were rated as less arousing when observed for 100 ms compared to 1,000 ms. There was no significant difference between the arousal ratings of neutral images (younger: *t*(124) = 1.72, *p* =.524, *d*_*z*_ = 0.15; older: *t*(114) = 0.43, *p* = 1.000, *d*_*z*_ = 0.04). Altogether, both younger and older adults rated emotional images as less extreme in arousal when images were observed for a shorter period of time compared to a longer period of time, and no difference in arousal for neutral images.

### Ratings About How Well an Image Was Seen

Although not part of our original analysis plan, we additionally considered how well images were seen in respect to the difference experimental conditions. We initially chose to ask participants how well they saw each image to exclude trials in which they could not see it. This ensured that image ratings would not include trials when participants could not see the image at all, and therefore better represent ratings for images related to the speed of exposure, rather than being seen or not seen per se. As we note above, when those trials remained in the dataset, their inclusion made little difference to the patterns we observed in the analyses above (see *Supplementary Material*). However, in an additional analysis stream, we chose to use participants’ ratings of how well they saw an image as a variable of interest to see if it varied between age group, exposure time, or age. To do this, we used participants’ reports of how well they saw an image as a continuous, dependent variable, and included all trials for all participants for these analyses.

There was a strong correlation between how well an image was seen at short (100 ms) and long (1,000 ms) exposure times in both younger (*r* =.575, *p* <.001) and older adults (*r* =.572, *p* <.001). In other words, if participants reported finding it difficult to see particular images when they were shown briefly, participants also reported finding it difficult to see the same images when they were shown for a longer time.

A 3 (image type: positive, neutral, and negative) × 2 (exposure time: 100 ms, 1,000 ms) × 2 (age: younger and older) mixed ANOVA further revealed that how well images were seen was related to the factors of this experiment (see Table 3). There was a main effect of age group, *F*(1, 238) = 6.65, *p* =.010, *η*^*2*^_*p*_ =.03; older adults reported having a harder time seeing images than younger adults overall. There was also a main effect of exposure time, *F*(1, 238) = 2351.25, *p* <.001, *η*^*2*^_*p*_ =.91, with images reported to be harder to see on short (100 ms) exposure trials compared to long (1,000 ms) trials. A main effect of image type, *F*(1.94, 461.35) = 87.59, *p* <.001, *η*^*2*^_*p*_ =.27, along with descriptive statistics further revealed that, in general, negative images were harder to see than the other image types in both younger and older adults.

**Table 3:** Means and 95% Confidence Intervals of image seen ratings

Image type	Exposure time	Image seen ratings			
		Younger adults		Older adults	
		*M*	*95% CI*	*M*	*95% CI*
Positive	100 ms	2.81	[2.72, 2.89]	2.44	[2.35, 2.53]
	1,000 ms	3.73	[3.68, 3.78]	3.87	[3.82, 3.92]
Neutral	100 ms	2.73	[2.64, 2.82]	2.49	[2.40, 2.58]
	1,000 ms	3.66	[3.60, 3.71]	3.86	[3.81, 3.92]
Negative	100 ms	2.58	[2.51, 2.66]	2.18	[2.10, 2.26]
	1,000 ms	3.71	[3.66, 3.76]	3.76	[3.70, 3.81]

There was also an age group × exposure time interaction, *F*(1, 238) = 86.76, *p* <.001, *η*^*2*^_*p*_ =.27, with older adults affected by exposure time more than younger adults, and an age group × image type interaction, *F*(1.94, 461.35) = 21.28, *p* <.001, *η*^*2*^_*p*_ =.08, reflecting that younger and older adults differed in how well different image types were seen. A significant exposure type × image type interaction, *F*(1.98, 471.45) = 50.87, *p* <.001, *η*^*2*^_*p*_ =.18, indicated that the difference in being seen between 100 ms and 1,000 ms varied significantly across the different image types. However, there was no significant three way image type × exposure time × age group interaction, *F*(1.98, 471.45) = 1.14, *p* =.322, *η*^*2*^_*p*_ =.005, indicating that the relative relationships between exposure time and image type were similar across younger and older adults. To follow-up on the role of exposure time, subsequent *t*-tests revealed that in both younger and older adults, for all image types, there was a significant difference in ratings about how well participants could see images at 100 ms compared to at 1,000 ms (*ts* > 22.50, *ps* <.001); across all conditions, images were harder to see when shown briefly.

### The Role of Image Properties on Ratings at Different Exposure Times

In a final exploratory analysis, we investigated whether image properties could predict differences between fast and slow ratings. Since younger and older adults rated images overall quite similarly, we chose to collapse across age groups for these analyses. Regardless of image type and regardless of age group, we calculated the mean long (1,000 ms) and slow (100 ms) ratings of valence, arousal, and how well an image was seen for each image. We then subtracted the mean ratings at 100 ms from mean ratings at 1,000 ms (i.e., long minus short exposure time trials). In other words, we calculated three scores for each image to represent differences in ratings on long versus short exposure times: a difference score for valence ratings, a difference score for arousal ratings, and a difference score for how well an image was seen. Pearson correlations between these difference scores and all individual image properties are available in our *Supplementary Material.* However, to examine the combination of features that were predictive, rather than individual features alone, we chose to regress the image difference scores onto image properties using three separate bidirectional stepwise regression models, which we analyzed using the *step()* function in R and displayed using the *sjPlot* package in R. Both forward selection and backward elimination were used to identify a model with a minimal Akaike Information Criterion (AIC) to select the predictors that provide the best fit with the most simple model. The regression model outputs, including contributions of each predictor, are available in Table 4.

**Table 4:** Stepwise regressions with image features

(a) Valence rating differences			(b) Arousal rating differences			(c) Seen rating differences		
*Predictors*	*Estimates*	*p*	*Predictors*	*Estimates*	*p*	*Predictors*	*Estimates*	*p*
(Intercept)	5.23	.143	(Intercept)	− 0.37	.275	(Intercept)	2.29	.009*
RAvg	− 0.11	.031*	RAvg	0.03	.016*	QhF	7.41e-03	.024*
Ef70	0.06	.018*	GAvg	− 0.02	.046*	memorability	− 1.49	.147
entropy	0.35	.079	Ef30	0.20	.126	conv3	− 4.47e-05	.033*
mean luminance	23.00	.038*	Ef50	− 0.15	.074	conv5	− 2.35e-04	.002*
conv1	− 9.42e-05	.031*	Ef90	0.01	.141	fc7	2.06e-03	<.001*
conv3	− 1.36e-04	.011*	conv1	2.11e-05	.060	fc8	2.41e-03	.089
			fc6	− 5.67e-04	.061			
			fc7	2.41e-03	.019*			
			fc8	− 7.29e-03	.001*			
*R*^2^/*R*^2^ adjusted:.127/.068			*R*^2^/*R*^2^ adjusted:.257/.179			*R*^2^/*R*^2^ adjusted:.390/.348		

Exploratory stepwise regression (forward and backward selection) outputs with image features as predictors for long (1,000 ms) minus short (100 ms) mean ratings of (a) valence, (b) arousal, and (c) self-reported ability to see an image. * denotes *p* <.05

Note that our goal was to examine how image features predicted the difference in ratings between short and long exposure durations. Our *Supplementary Materials* also include exploratory stepwise regressions for younger and older adults separately and for short and long exposures separately, for readers interested.

For the valence difference scores, the predictors that were selected based on the lowest model AIC value (AIC = 11.06) were RAvg, Ef70, entropy, mean luminance, conv1 and conv3. The model was a weak fit for predicting the valence difference scores between fast and slow ratings, *R*^2^ =.127, adjusted *R*^2^ =.068. Significant predictors of the valence differences included the color red, spatial frequency in the 70% band, luminance, and two early convolution layers (conv1 and conv3) of AlexNet (see Table 4). For arousal differences, the stepwise multiple regression model with the lowest AIC value (AIC =  − 102.51) included RAvg, GAvg, Ef30, Ef50, Ef90, conv1, fc6, fc7, and fc8. This model was a moderate fit of the data; *R*^2^ =.257, adjusted *R*^2^ =.179. Red and green color and higher AlexNet layers (fc7 and fc8) were significant predictors in this model (Table 4). Thus, for both valence and arousal rating differences between fast and slow exposures, image properties could predict some of the variance, and properties related to color and AlexNet were significant predictors both of these stepwise models. The final stepwise model for differences in how well an image was seen included QhF, memnet, conv3, conv5, fc7, and fc8. This model predicted 39% of the variance, *R*^2^ =.390, adjusted *R*^2^ =.348, indicating a good model fit. The intercept was significant, as were the measures of high spatial frequency, memorability, and several layers of AlexNet (convolution layers 3 and 5 and fully connected layers 7 and 8; see Table 4).

## Discussion

Prior work has examined behavioral effects of emotional stimuli presented at fast speeds (e.g., Kennedy & Most, 2015a, 2015b; Most et al., 2005; Wang et al., 2012), but little is known about how exposure time influences subjective ratings of emotions, especially across different age groups. We found a strong correlation between ratings of emotional stimuli at brief (100 ms) and longer (1,000 ms) exposure times, but found that both younger and older adults rated emotional images as less extreme along the dimensions of valence and arousal when those images were shown for a shorter compared longer period of time. Older adults were more impacted by the difference in exposure time and generally rated images as more positive in valence than younger adults. When the differences in ratings between exposure types were regressed onto image properties in an exploratory analysis, some image properties — especially related to color and layers in the neural net, AlexNet — significantly contributed to the models.

Our results add to existing work by demonstrating that although within 100 ms most emotional information was available for participant ratings, emotion ratings were blunted in intensity, suggesting that increased time may be involved in further developing those emotional experiences. This may align with theories of emotion that imply emotional experiences take time to construct (Barrett, 2017) or regulate (Gross, 2015). Additionally, both age groups rated neutral images shown for 1,000 ms as more positive than neutral images shown for 100 ms. While speculative, this is consistent with evidence that emotionally ambiguous images are rated as more positive in both younger and older adults when participants are encouraged to take more time to respond (Neta & Tong, 2016).

In the greater context of affective aging, older adults were especially affected by exposure time. Affective aging theories commonly implicate speed, such as slower processing speed (e.g., Salthouse, 2010) or age-related regulation strategies (e.g., Mather & Carstensen, 2005). In the current study, older adults rated images of all types as more positive than younger adults, suggesting that a positivity bias in aging persisted at fast speeds, resembling biases reported in similar rating tasks (Neiss et al., 2009; Neta & Tong, 2016). At the same time, it is notable that our design matched this previous research in the way images were shown for a set, short amount of time at both exposure times (100 ms and 1,000 ms) than research that tends to indicate a more negative bias (Backs et al., 2005; Grühn & Scheibe, 2008). Together these findings indicate that age-related positivity biases in image ratings occur quickly, but extended time or different rating task designs may reverse that bias, which provides a rich avenue for future research.

We originally included a measure of how well participants could see an image to be able to exclude trials when they did not see an image. This was important to ensure our ratings were true ratings, rather than including guesses when participants had not seen an image at all. However, when we used this measure as a dependent variable, we found that images shown quickly were more difficult to see, and images that were more difficult to see at short exposure times were also more difficult at longer exposure times. Negative images were most difficult to see; while the reason for this is yet unclear, it could be that negative images tend to show more complicated content.

When it came to image properties, our exploratory analyses suggested that some image properties can predict emotional experiences at different speeds. Factors that were predictive of valence and arousal rating differences between exposure times were color (especially red) and layers from AlexNet, which simulates levels of processing in the brain (Krizhevsky et al., 2017). The image properties that predicted whether an image was seen or not included high spatial frequency information, the memorability of the images, and several AlexNet layers, and this model was a stronger fit than those for valence and arousal differences. While preliminary, these results indicate that several image features contribute to the subjective ratings of emotional stimuli at varying speeds, especially how well an image can be seen, providing candidate mechanisms involved in the temporal dynamics of emotion perception.

Practically, our results offer guidance for future emotion research. Strong correlations between fast and slow exposures indicate that normative ratings of images remain informative, however, researchers should be cautioned when using emotional images that are less powerful if they are showing them briefly. Mildly emotional images based on normative ratings may be experienced in the spectrum of more emotionally neutral when presented quickly, and complex images that may be difficult to see at longer rates should be avoided for faster tasks.

Our study was not without limitations. Conducting our experiment online removed experimental control that can otherwise be achieved in the laboratory (Grootswagers, 2020), although we attempted to overcome this limitation by using millisecond sensitive software (Inquisit 6, 2021) and allowing participants to indicate if they did not see an image. Another possible, related, limitation is that we compared undergraduate Australian younger adults and American older adults. However, this limitation should be tempered in how age differences were as predicted and MTurk participants tend to have higher depressive symptoms and better cognitive abilities (McCredie & Morey, 2019; Ogletree & Katz, 2021), which makes their comparative positivity bias despite these differing sample characteristics.

Overall, our results suggest that exposure time can change the way that participants rate emotional images and that age can amplify these effects. This has implications in the speed in which emotional stimuli are processed, practical implications for researchers to consider when selecting stimuli or interpreting results, and inform how age and different exposure times can affect the intensity of emotional experiences. 

## Supplementary Information

Below is the link to the electronic supplementary material.

ESM 1 (56.6 KB DOCX)

## Additional Information

### Availability of Data and Materials

Experimental materials and anonymized data are available at https:/osf.io/vxuf8/
