# Supplementary material for: The Role of Exposure Time on the Subjective Ratings of Emotional Images in Younger and Older Adults
Source: Affect Sci. 2025 Oct 31;6(4):668–79. doi: 10.1007/s42761-025-00326-9 (PMC12894480; doi:10.1007/s42761-025-00326-9)
Supplement: Supplementary file 1 — (56.6 KB DOCX) [file 42761_2025_326_MOESM1_ESM.docx]

Supplemental Material: The role of exposure time on the subjective ratings of emotional images in younger and older adults

Briana L. Kennedy^1^, Jacqueline W. Tan^1^, Tijl Grootswagers^2,3^, & Steven B. Most^4^

^1^ School of Psychological Science, The University of Western Australia, Perth, Australia

^2^ The MARCS Institute for Brain, Behaviour and Development, Western Sydney University, Sydney, Australia

^3^ School of Computer, Data and Mathematical Sciences, Western Sydney University, Sydney, Australia

^4^ School of Psychology, UNSW Sydney, Sydney, Australia

Correspondence concerning this article should be addressed to:

Briana L. Kennedy

School of Psychological Science, University of Western Australia (M304)

35 Stirling Highway

Crawley WA 6009 Australia

Phone: +61 8 6488 3649

Email: briana.kennedy@uwa.edu.au

**Analyses with all trials included**

We chose to remove trials when participants reported that they did not see an image to ensure that the ratings reflected true ratings rather than guesses about an image they did not see. However, when we reincluded those trials in the dataset, results from the analyses were essentially the same. We report those results here for completeness.

For valence ratings, a 3 (image type: positive, neutral, and negative) × 2 (exposure time: 100ms, 1000ms) × 2 (age: younger and older) ANOVA revealed significant main effects of image type, *F*(1.56, 371.31) = 2713.59, *p* < .001, *η^2^_p_* = .92, exposure time, *F*(1, 238) = 15.35, *p* = .005, *η^2^_p_* = .06, and age, *F*(1, 238) = 29.92, *p* < .001, *η^2^_p_* = .11. All interactions between factors were also significant, including the image type × age, *F*(1.56, 371.31) = 6.48, *p* = .004, *η^2^_p_* = .03, image type × exposure time, *F*(1.78, 424.64) = 1096.05, *p* < .001, *η^2^_p_* = .82, exposure time × age, *F*(1, 238) = 26.79, *p* < .001, *η^2^_p_* = .10, and three-way image type × exposure time × age, *F*(1.78, 424.64) = 115.97, *p* < .001, *η^2^_p_* = .33.

For arousal ratings, a 3 (image type: positive, neutral, and negative) × 2 (exposure time: 100ms, 1000ms) × 2 (age: younger and older) ANOVA also revealed main effects for image type, *F*(1.55, 369.88) = 493.09, *p* < .001, *η^2^_p_* = .67, and exposure time, *F*(1, 238) = 37.11, *p* < .001, *η^2^_p_* = .92, but not age, *F*(1, 238) = 0.76, *p* = .384, *η^2^_p_* < .01. Similarly, there was no significant interaction between image type × age, *F*(1.55, 369.88) = 1.08, *p* = .327, *η^2^_p_* < .01, however there were significant interactions between image type × exposure time, *F*(1.88, 447.75) = 225.34, *p* < .001, *η^2^_p_* = .49, exposure time × age, *F*(1, 238) = 364.72, *p* < .001, *η^2^_p_* = .13, and the three-way interaction between image type × exposure time × age, *F*(1.88, 447.75) = 29.72, *p* < .001, *η^2^_p_* = .11.

**Pearson correlations between image properties**

We ran regression analyses to examine the relationship between image features and ratings on long (1000ms) versus short (100ms) trials. Table S1 presents Pearson correlations between each of the image features and difference scores in ratings.

**Table S1.**

*Pearson Correlations with Image Features*

| **Variable** | ***1*** | ***2*** | ***3*** | ***4*** | ***5*** | ***6*** | ***7*** | ***8*** | ***9*** | ***10*** | ***11*** | ***12*** | ***13*** | ***14*** | ***15*** | ***16*** | ***17*** | ***18*** | ***19*** | ***20*** | ***21*** | ***22*** | ***23*** | ***24*** |
| --- | --- | --- | --- | --- | --- | --- | --- | --- | --- | --- | --- | --- | --- | --- | --- | --- | --- | --- | --- | --- | --- | --- | --- | --- |
| *1. ValenceMean_diff* |  |  |  |  |  |  |  |  |  |  |  |  |  |  |  |  |  |  |  |  |  |  |  |  |
| *2. ArousalMean_diff* | -.49** |  |  |  |  |  |  |  |  |  |  |  |  |  |  |  |  |  |  |  |  |  |  |  |
| *3. SeenMean_diff* | -.09 | .3* |  |  |  |  |  |  |  |  |  |  |  |  |  |  |  |  |  |  |  |  |  |  |
| *4. RAvg* | -.04 | .21* | -.16 |  |  |  |  |  |  |  |  |  |  |  |  |  |  |  |  |  |  |  |  |  |
| *5. GAvg* | -.06 | .19 | -.14 | .76** |  |  |  |  |  |  |  |  |  |  |  |  |  |  |  |  |  |  |  |  |
| *6. BAvg* | -.03 | .16 | -.05 | .43** | .86** |  |  |  |  |  |  |  |  |  |  |  |  |  |  |  |  |  |  |  |
| *7. Ef10* | .16 | .07 | .04 | .08 | .19 | .2 |  |  |  |  |  |  |  |  |  |  |  |  |  |  |  |  |  |  |
| *8. Ef30* | .13 | .05 | .19 | .02 | .13 | .14 | .64** |  |  |  |  |  |  |  |  |  |  |  |  |  |  |  |  |  |
| *9. Ef50* | .14 | .02 | .28* | .01 | .12 | .16 | .52** | .92** |  |  |  |  |  |  |  |  |  |  |  |  |  |  |  |  |
| *1. Ef70* | .13 | .04 | .29* | -.01 | .13 | .18 | .46** | .82** | .92** |  |  |  |  |  |  |  |  |  |  |  |  |  |  |  |
| *11. Ef90* | .08 | .08 | .33** | -.03 | .15 | .24* | .48** | .77** | .87** | .94** |  |  |  |  |  |  |  |  |  |  |  |  |  |  |
| *12. QhF* | .12 | .06 | .33** | -.01 | .13 | .19 | .51** | .83** | .93** | .97** | .97** |  |  |  |  |  |  |  |  |  |  |  |  |  |
| *13. entropy* | -.03 | .11 | -.02 | .63** | .65** | .54** | .2* | .15 | .18 | .14 | .14 | .14 |  |  |  |  |  |  |  |  |  |  |  |  |
| *14. mean_luminance* | -.05 | .21* | -.13 | .84** | .98** | .84** | .18 | .1 | .1 | .1 | .12 | .1 | .69** |  |  |  |  |  |  |  |  |  |  |  |
| *15. rms_contrast* | -.05 | .12 | -.19 | .47** | .48** | .35** | -.14 | -.26* | -.2* | -.2* | -.2 | -.21* | .16 | .48** |  |  |  |  |  |  |  |  |  |  |
| *16. memorability* | .11 | -.2 | -.38** | -.09 | -.12 | -.13 | -.18 | -.26* | -.3* | -.33** | -.37** | -.36** | -.25* | -.13 | .04 |  |  |  |  |  |  |  |  |  |
| *17. conv1* | -.03 | 0 | .07 | -.39** | .28* | .64** | .17 | .14 | .15 | .2 | .28* | .21* | .04 | .18 | -0 | -.05 |  |  |  |  |  |  |  |  |
| *18. conv2* | .08 | -.18 | -.19 | -.45** | -.54** | -.46** | -.32* | -.45** | -.48** | -.49** | -.53** | -.5** | -.68** | -.54** | -.2 | .52** | -.12 |  |  |  |  |  |  |  |
| *19. conv3* | -.14 | .21* | .18 | .3* | .41** | .37** | .14 | .29* | .31* | .35** | .36** | .34** | .7** | .41** | -.1 | -.51** | .16 | -.77** |  |  |  |  |  |  |
| *20. conv4* | .1 | -.21* | -.29* | -.19 | -.35** | -.36** | -.21* | -.4** | -.43** | -.46** | -.48** | -.47** | -.57** | -.34** | .15 | .61** | -.24* | .81** | -.94** |  |  |  |  |  |
| *21. conv5* | .08 | -.27* | -.41** | -.09 | -.22* | -.25* | -.19 | -.35** | -.38** | -.4** | -.44** | -.42** | -.41** | -.22* | .16 | .65** | -.21* | .69** | -.82** | .91** |  |  |  |  |
| *22. fc6* | -.13 | -.01 | .23* | -.24* | 0 | .22* | 0 | 0 | .05 | .1 | .17 | .13 | -.11 | -.02 | -.2 | -.28* | .41** | -.04 | .07 | -.19 | -.14 |  |  |  |
| *23. fc7* | -.16 | .13 | .32* | -.21* | -.18 | -.1 | -.39** | -.22* | -.12 | -.13 | -.08 | -.1 | -.22* | -.19 | -.1 | -.02 | .06 | .15 | -.11 | .09 | 0 | .45** |  |  |
| *24. fc8* | .13 | -.32* | -.07 | .08 | -.18 | -.26* | -.08 | -.1 | -.08 | -.13 | -.16 | -.14 | -.02 | -.13 | .15 | .33** | -.36** | .19 | -.39** | .39** | .41** | -.29* | -.1 |  |

*Note. * denotes p < .05. **denotes p < .001*

**Stepwise Regressions with Image Means in Younger and Older Adults Separately**

We chose to collapse across age groups in our main exploratory analysis of image features. However, in a supplementary analysis, we conducted the same stepwise regressions in younger and older adults separately (Table S2).

**Table S2**

*Stepwise regressions with image features in younger and older adults separately*

| (a) Valence Rating Differences | | | (b) Valence Rating Differences | | |
| --- | --- | --- | --- | --- | --- |
| *Predictors* | *Estimates* | *p* | *Predictors* | *Estimates* | *p* |
| (Intercept) | 7.07 | .013* | (Intercept) | -4.62 | .010* |
| RAvg | -0.11 | .009* | Ef70 | 0.07 | 0.058 |
| Ef70 | 0.05 | .019* | entropy | 0.63 | .013* |
| mean luminance | 23.27 | .009* | conv2 | 6.22e-05 | .076 |
| conv1 | -9.48e-05 | .007* | conv3 | -4.55e-04 | .007* |
| conv3 | -6.51e-05 | .042* | conv4 | -6.74e-4 | .026* |
|  |  |  | fc6 | -1.08e-03 | .080 |
|  |  |  |  |  |  |
|  |  |  |  |  |  |
| R^2^ / R^2^ adjusted: .146 / .099 | | | R^2^ / R^2^ adjusted: .144 / .087 | | |
| (c) Arousal Rating Differences | | | (d) Arousal Rating Differences | | |
| *Predictors* | *Estimates* | *p* | *Predictors* | *Estimates* | *p* |
| (Intercept) | -4.28 | .020* | (Intercept) | -2.17 | .007* |
| RAvg | 0.07 | .016* | RAvg | 0.01 | .036* |
| mean luminance | -13.03 | .023* | Ef30 | 0.44 | .030* |
| conv1 | 4.96e-05 | .028* | Ef50 | -0.25 | .044* |
| conv3 | -8.84e-05 | .119 | Ef90 | 0.02 | .107 |
| conv4 | -1.84e-04 | .058 | rms contrast | 4.17 | .029* |
| fc6 | -5.94e-04 | .044* | conv2 | 5.53e-05 | .006* |
| fc7 | 2.17e-03 | .022* | conv5 | -4.44e-04 | .001* |
| fc8 | -0.01 | .001* | fc7 | 1.95e-03 | .149 |
| R^2^ / R^2^ adjusted: .281 / .215 | | | R^2^ / R^2^ adjusted: .208 / .135 | | |
| (e) Seen Rating Differences | | | (f) Seen Rating Differences | | |
| *Predictors* | *Estimates* | *p* | *Predictors* | *Estimates* | *p* |
| (Intercept) | 3.03 | <.001* | (Intercept) | 1.6 | <.001* |
| QhF | 0.01 | .004* | RAvg | -2.22e-03 | .106 |
| memorability | -2.7 | .002* | QhF | 0.01 | .032* |
| fc7 | 1.82e-03 | .001* | conv4 | 1.61e-04 | .007* |
|  |  |  | conv5 | -4.73e-04 | <.001* |
|  |  |  | fc7 | 2.10e-03 | .001* |
|  |  |  | fc8 | 3.39e-03 | .030* |
|  |  |  |  |  |  |
|  |  |  |  |  |  |
| R^2^ / R^2^ adjusted: .296 / .273 | | | R^2^ / R^2^ adjusted: .427 / .388 | | |

*Note.* Exploratory stepwise regression (forward and backward selection) outputs with image features as predictors for mean ratings of (a) valence for younger adults, (b) valence for older adults, (c) arousal for younger adults, (d) arousal for older adults, (e) self-reported ability to see an image for younger adults, and (f) self-reported ability to see an image for older adults. Valence and arousal did not include trials when the image was unseen, while self-reported ability to see an image included all trials regardless of whether an image was unseen. * denotes *p* < .05.

Image features were similarly predictive for younger adults and older adults in valence ratings, arousal ratings, and seen ratings. Notably, many of the same features were similar in younger and older adults. These data are exploratory and any differences between younger and older adults should be interpreted with appropriate caution. Nevertheless, future research may benefit from exploring some of these different features in the way they impact emotionality ratings in younger versus older adults, for example, how spatial frequency may impact older adults’ but not younger adults’ ratings of arousal, or how convolution layers can predict older adults’ but not younger adults’ ability to see images at these speeds.

**Stepwise Regressions with Image Means on Fast and Slow Trials Separately**

Our goal was to examine how image features predicted the difference in ratings between fast and slow trials. However, in supplemental analyses, we conducted exploratory stepwise regressions for fast and slow trials separately on mean ratings of valence, arousal, and self-reported ability for an image to be seen (Table S3).

**Table S3**

*Stepwise regressions with image features for mean valence and arousal ratings on 100ms and 1000ms trials*

| (a) Valence on 100ms Trials | | | (b) Valence on 1000ms Trials | | |
| --- | --- | --- | --- | --- | --- |
| *Predictors* | *Estimates* | *p* | *Predictors* | *Estimates* | *p* |
| (Intercept) | -1.66 | .347 | (Intercept) | -1.32 | .573 |
| BAvg | 9.26e-03 | .110 | entropy | 0.96 | .006* |
| Ef70 | 0.17 | .156 | conv3 | -6.74e-04 | .008* |
| QhF | -0.06 | .173 | conv4 | -7.20e-04 | .057 |
| entropy | 0.84 | .002* | fc7 | -8.97e-03 | .008* |
| conv2 | 5.82e-05 | .093 |  |  |  |
| conv3 | -6.09e-04 | .001* |  |  |  |
| conv4 | -9.33e-04 | .003* |  |  |  |
| fc6 | -1.53e-03 | .037* |  |  |  |
| fc7 | -3.94e-03 | .095 |  |  |  |
| R^2^ / R^2^ adjusted: .325 / .255 | | | R^2^ / R^2^ adjusted: .186 / .150 | | |
| (c) Arousal on 100ms Trials | | | (d) Arousal on 1000ms Trials | | |
| *Predictors* | *Estimates* | *p* | *Predictors* | *Estimates* | *p* |
| (Intercept) | 2.62 | .275 | (Intercept) | 0.70 | .534 |
| GAvg | -6.16e-02 | .001* | RAvg | 3.15e-02 | .001* |
| Ef30 | 0.72 | .002* | GAvg | -4.43e-02 | .013* |
| Ef50 | -0.41 | .008* | BAvg | 1.97e-02 | .071 |
| Ef70 | 9.56e-02 | .067 | Ef30 | 0.96 | .002* |
| mean luminance | 12.2 | .001* | Ef50 | -0.54 | .010* |
| rms contrast | 6.85 | .002* | Ef70 | 0.11 | .109 |
| memorability | 3.65 | .172 | rms contrast | 8.71 | .003* |
| conv2 | 5.49e-05 | .010* | conv2 | 7.87e-05 | .007* |
| conv5 | -4.31e-04 | .009* | conv5 | -5.74e-04 | .006* |
| fc7 | 2.61e-03 | .076 | fc7 | 4.09e-03 | .040* |
| fc8 | -7.28e-03 | .062 | fc8 | -1.26e-02 | .017* |
| R^2^ / R^2^ adjusted: .302 / .210 | | | R^2^ / R^2^ adjusted: .335 / .248 | | |
| (e) Seen on 100ms Trials | | | (f) Seen on 1000ms Trials | | |
| *Predictors* | *Estimates* | *p* | *Predictors* | *Estimates* | *p* |
| (Intercept) | 1.50 | .179 | (Intercept) | 3.89 | <.001* |
| GAvg | 4.56e-03 | .026* | RAvg | -1.06e-02 | .087 |
| QhF | -9.46e-03 | .014* | Ef30 | 5.25e-02 | .056 |
| entropy | -0.12 | .118 | Ef50 | -2.73e-02 | .049* |
| memorability | 1.82 | .127 | mean luminance | 2.44 | .063 |
| conv3 | 6.67e-05 | .048* | memorability | 0.59 | .087 |
| conv5 | 2.61e-04 | .005* | conv1 | -9.32e-06 | .070 |
| fc7 | -2.10e-03 | .001* | conv4 | 1.52e-05 | .137 |
| R^2^ / R^2^ adjusted: .405 / .358 | | | R^2^ / R^2^ adjusted: .239 / .179 | | |

*Note.* Exploratory stepwise regression (forward and backward selection) outputs with image features as predictors for mean ratings of (a) valence on short (100ms) trials, (b) valence on long (1000ms) trials, (c) arousal on short (100ms) trials, (d) arousal on long (1000ms) trials, (e) self-reported ability to see an image on short (100ms) trials, and (f) self-reported ability to see an image on long (1000ms) trials. Like regression analyses reported in the main manuscript with difference scores, mean ratings were averaged across younger and older adults. Valence and arousal did not include trials when the image was unseen, while self-reported ability to see an image included all trials regardless of whether an image was unseen. * denotes *p* < .05.

These stepwise regressions indicated that image features were moderately predictive for each of the rating types, with R^2^ adjusted values between 15% and 35.8%. While beyond the objectives of the current study, future research should continue to examine how image properties relate to the way emotional stimuli are experienced.
